# The relationship between core stability and athletic performance in female university athletes

**DOI:** 10.17159/2078-516X/2021/v33i1a10825

**Published:** 2021-08-23

**Authors:** M de Bruin, D Coetzee, R Schall

**Affiliations:** 1 Department of Exercise and Sport Sciences, Faculty of Health Sciences, University of the Free State, Bloemfontein, South Africa; 2 Department of Mathematical Statistics and Actuarial Science, Faculty of Natural and Agricultural Sciences, University of the Free State, Bloemfontein, South Africa

**Keywords:** hockey, netball, running, soccer, tennis, functional stability

## Abstract

**Background:**

Questions remain as to whether core stability represents single or multiple components, how to assess core stability, and if a relationship exists with athletic performance in different sporting codes.

**Objectives:**

To investigate the relationship between core stability and athletic performance in female university athletes.

**Methods:**

Eighty-three female athletes (hockey, netball, running, soccer and tennis) participated in this quantitative, cross-sectional study. The isometric back extension (IBE), lateral flexion (LF) and abdominal flexion (AF) tests were used to measure core strength and endurance. The core stability grading system using a pressure biofeedback unit was applied to measure core neuromuscular control (NMC). Athletic performance was assessed using the 40 m sprint, T-test, vertical jump (VJ) and the medicine ball chest throw (MBCT). Correlations between the core stability tests and the athletic performance tests were determined overall and separately by sport. The effect of core stability on athletic performance was analysed using ANCOVA.

**Results:**

Overall for all sports, most correlations were weak (r=0.10–0.39), although there was a very strong correlation between LF (strength) and VJ (r=0.90). When the sports were considered separately, there were moderate correlations (r=0.40–0.69) between core strength, endurance and motor control with certain athletic performance tests in all five sport codes. In runners, strong correlations (r=0.70–0.89) were observed between AF (endurance) and VJ, and in tennis players between IBE (strength) and the sprint.

**Conclusion:**

Correlations were found between core stability and athletic performance, although most correlations were negligible or weak. Athletic performance in different sport codes is associated with different components of core stability.

Core stability has been studied for more than 40 years and has become fundamental to training programmes for performance enhancement in diverse sporting codes.^[1]^ However, the two fundamental concepts of core strength and core stability have generally not been distinguished.^[1]^

Akuthota and Nadler defined core strength as the involvement of the anatomical structures around the lumbar spine in the maintenance of functional stability.^[2]^ This definition differs from the usual notion of strength in athletes proposed by Lehman as a maximum force produced by a muscle group at a certain velocity.^[3]^ The training of core strength includes several repetitions performed at a high load with core muscle tension.^[4]^ Core strength is vital for sport performance and should be considered as an important component to determine core stability.^[5]^ Core endurance can be defined as the potential of the core muscles to avoid fatigue during continuous low-load activities.^[6]^ Core motor control is the activation of core muscles in a specific task controlled by the central nervous system.^[6]^ Consensus on how these different components should be defined and assessed is lacking. No gold-standard measurement has been described or suggested for the evaluation of core stability.^[7]^ Therefore, core stability assessment should consist of a battery of tests that evaluates various components of the core, depending on the demand of the task.^[8]^

Knowledge of functional core stability has led to the ability to classify and identify the components that affect core muscle function. The core muscles are important for dynamic stabilisation.^[9]^ Research on core stability exercise programmes and the associated improvement of athletic performance is limited.^[1]^ Sharrock et al.^[5]^ concluded from a pilot study that future research should attempt to establish the sub-categories involved in core stability and identify which are most important for individual sport codes. The aim of this study was to evaluate the relationship between core stability, including the sub-categories of strength, endurance and neuromuscular control (NMC), and athletic performance among female university athletes in hockey, netball, running, soccer and tennis.

## Methods

### Participants and study design

In the 2018/2019 season, 122 female athletes were members of the first hockey, netball, running, soccer and tennis teams at the University of the Free State (UFS) in Bloemfontein, South Africa. All these athletes were invited to participate in the study. Subjects were excluded from the study if they (i) had an acute, medically diagnosed injury that required medical treatment during the preceding three months, (ii) had an illness on the day of testing, or (iii) refused informed consent to participate in the study.

Data were collected and recorded using a scoring sheet. The scoring sheet indicated the core stability and athletic performance tests, and the sequence of testing. Testing of the various sport teams took place on different days as part of their periodisation in the off-season and pre-season, respectively. One week before testing, the participants were informed that on the day of testing they were not allowed to exercise. On the day of the testing, the participants did a 10-minute warm-up on a cycle ergometer and performed dynamic stretches.

### Ethical considerations

Approval to conduct the research was granted by the Health Sciences Research Ethics Committee (HSREC) of the Faculty of Health Sciences, University of the Free State (reference number UFS-HSD2019/0447/2506). All participants provided their informed consent.

### Core stability testing

#### Core strength tests

As described by Saeterbakken et al.,^[10]^ the core strength of the global core muscles was measured using the Biering-Sørensen tests, which included isometric back extension (IBE), lateral flexion (LF) and abdominal flexion (AF). The participants were required to contract their global core muscles to their maximum for three seconds. The explosive power output of the maximum volunteered contraction was measured using a Tendo sports machine (Tendo Sports Machines; Trencin, Slovak Republic). Each of the three respective core strength tests were performed three times with a one-minute rest between the attempts and three minutes of rest before performing the next test. The greatest mean force output in Newtons (N) over three seconds for each test (IBE, LF and AF) was used in further analyses.

The Biering-Sørensen IBE test was assessed in a prone position on an exercise bed. The participant had to hold the prone position until failure.^[11]^ The edge of the iliac crest was positioned on the tip of the bed while the arms were crossed over the chest. Lastly, the body was in a straight position with the feet secured to the bed by the ankles.^[11]^

During the LF test, the participant lay down horizontally with their legs and hip relaxed on the bed. The participants were not allowed to rest on their elbows while their feet were tied with a strap to the bed across their ankles. Only the dominant side was assessed (facing upwards) and the non-dominant arm was crossed over the chest.^[10]^

For the AF test position, the participant had to hold a 45° angle between their hips and the bed and the hips and knees were bent at 90° angle. The spine had to be held upright while the arms were crossed over the chest and their feet secured to the bed.^[11]^

#### Core endurance tests

The same positions used to measure the core strength of the global muscles were used to assess the core endurance of the global muscles. To determine the endurance of the global core muscles, participants were required to contract these muscles to the maximum for as long as they could. A stopwatch was used to determine how long they could hold this contraction. A reliability coefficient of 0.97–0.99 has been reported for the Bering-Sørensen method.^[11]^ All tests were terminated when the participant fell below the test position.

#### Core neuromuscular control (NMC) tests

Core neuromuscular control (NMC) was assessed using a Welch Allyn FlexiPort (Hill-Rom Holdings Inc.; Chicago IL, USA) pressure biofeedback unit. This was able to determine changes in pressure while the local and global core muscles aimed to stabilise the trunk during low-load limb movement.^[12]^ An interclass correlation coefficient (ICC) of 0.91 for test-retest reliability of the pressure biofeedback unit has been reported.^[12]^ The Wisbey-Roth core stability grading system was used to classify the motor control component of core stability.^[13]^

The participant lay in a supine position on an exercise bed. A pressure biofeedback unit was placed below the lower back and inflated to 40 millimetres of mercury (mmHg). The participant was instructed to maintain the pressure on the gauge while breathing regularly. This was considered as a grade 1 on the Wisbey-Roth core stability grading system.^[13]^ If the participant failed to maintain the pressure they scored grade 0. The participant obtained a grade 2 if they were able to maintain the pressure on the gauge while performing single leg slides of the limbs. A grade 3 was scored if the participant was able to maintain the pressure while performing slow movements of the trunk. If they were able to maintain the pressure on the gauge while performing fast movements of the limbs, a grade 4 was recorded and a grade 5 was obtained with fast movements of the trunk and limbs against resistance. Changes in the pressure greater than 10 mmHg were indicative of diminished core motor control. The next level was only determined only after the previous level had been successfully completed.^[13]^

Each activity level represented a level of core motor control.^[14]^ The participant was allowed one trial on each level and was only progressed to the next level after successful core motor control of the previous level. Scoring was the highest completed level where the instructed task was successfully completed with a change of less than 10 mmHg on the pressure biofeedback unit with a normal breathing pattern.^[15]^

### Athletic performance testing

#### Agility test

The T-test (ICC=0.97)^[16]^ was conducted to assess the participant’s agility in a forward, backward and lateral direction. The agility and direction changes performed during a T-test are used in a wide variety of sporting codes. Optimal agility is critical to complete the course and change of direction within the shortest amount of time.^[5]^

The time of the T-test (in seconds) was recorded using a stopwatch. Four cones were used as markers for the T-test. On the cue of the timer, the participant sprinted 10 m from the start in a forward direction to touch the base of the cone, then side-shuffled 5 m to the left to touch the base of the cone. The participant then changed direction to the right and shuffled 10 m to touch the base of the cone, changed direction again to the left and shuffled 5 m to touch the base of the cone, and then ran 10 m backwards to the start. Time was stopped when they passed the start. The participants completed three rounds and the best time to the nearest 0.1 seconds was recorded.

#### Speed test

The 40 m sprint test (sprint) (ICC=0.85)^[17]^ was used to assess the participant’s lower extremity explosive power and speed.^[18]^ Participants had to cover a distance of 40 m as fast as possible. The time was recorded from the first movement of the extremities and terminated when the participant crossed the line. The participants completed three rounds after which the fastest time to the nearest 0.1 seconds was recorded.

#### Lower extremity explosive power test

The vertical jump (VJ) was used to assess lower extremity explosive power. Sports such as netball, soccer and tennis require good explosive power of the lower extremities to jump specific heights. The Vertec (Rogue Fitness; Columbus OH, USA) VJ tester (ICC=0.99)^[19]^ was used to determine the VJ height. The participant stood facing the wall and reached up with both hands. The standing reach distance was recorded at the top of the fingertips while the participant stretched their arms above their head, keeping their feet flat on the ground. The participant was instructed to stand perpendicular to the Vertec with their body weight equally spread between the legs and feet and with the dominant side facing the wall. The participant was not allowed to perform a double bounce before the jump. The participant was allowed to bend the knees and then jump from both feet as high as possible and touch the Vertec with the dominant hand. The participants completed three trials and the best height to the nearest 0.1 cm was recorded.

#### Upper extremity explosive power test

The medicine ball chest throw (MBCT) (ICC=0.87–0.95)^[20]^ was used to assess upper extremity strength and explosive power.^[5]^ Many sports require overhead activities such as throwing a ball or catching an object, and a good level of upper extremity explosive power is required for optimal performance during these movements.^[5]^

The participant was instructed to stand in a kneeling position with knees bent at 90° and both hips in full extension. A distance of 10 m was measured out using a measuring tape. The 3 kg medicine ball was held with both hands in front of the chest. When the participant was ready, they could throw the ball vigorously as far forward as possible without falling forward or rocking back to gain momentum before the throw. Each participant was granted a practice trial to ensure they understood how to perform the movement. If the movement was carried out properly without compensatory or trick movements, the distance of the first bounce was measured. The participants completed three trials and the best distance to the nearest 0.1 m was recorded.

### Statistical analysis

Data were captured on a Microsoft Excel (Microsoft Office 2016) spreadsheet. Further analysis was performed using the SAS statistical software (SAS Institute Inc.; Cary, NC).

Data on core stability, namely IBE (strength), LF (strength), AF (strength), IBE (endurance), LF (endurance), AF (endurance), and core neuromotor control (NMC), and for the four tests of athletic performance (sprint, T-test, VJ and MBCT), were available for 83 athletes. The data on core strength, core endurance and core motor control were summarised using descriptive statistics. To assess the effect of core stability on athletic performance, both pairwise correlations and analysis of covariance (ANCOVA) were carried out.

#### Correlations

As described by Scharrock et al.,^[5]^ Pearson correlation coefficients and associated p-values were calculated between the characteristics of core strength, core endurance and core motor control and the four tests of athletic performance. This was done overall for all participants and separately for each sport. Correlations were referred to as ’negligible’ if their absolute value was in the range 0.00–0.10, ’weak’ (0.11–0.39), ’moderate’ (0.40–0.69), ’strong’ (0.70–0.89) and ’very strong ’ (0.90–1.00).^[21]^

#### ANCOVA

The four characteristics of athletic performance (40 m sprint, T-test, VJ and MBCT) were analysed using Analysis of Covariance (ANCOVA). In each case, the ANCOVA model fitted the characteristics of core stability IBE (strength), LF (strength), AF (strength), IBE (endurance), LF (endurance), AF (endurance) and core NMC as covariates. In addition, the ’type of sport’ was included since this factor potentially affects athletic performance as measured in the current study.

Initially, the full ANCOVA model was fitted with all independent variables listed above. Furthermore, backward model selection was performed as follows: starting with the full model fitting all the above variables, while at each selection step that variable was chosen for exclusion from the model whose exclusion from the model achieved the largest increase in the Schwarz Bayesian Information Criterion (SBC). For each assessment of athletic performance, the results of the final model selected by the SBC are reported here, together with estimates of the regression slopes and associated p-values.

## Results

Eighty-three female student athletes from the UFS participated in this study. The athletes represented hockey (n=24), netball (n=16), running (n=11), soccer (n=15) and tennis (n=17). Table 1 presents the descriptive statistics for the characteristics of core strength and core endurance (overall and by the type of sport). Similarly, Table 2 summarises the descriptive statistics with respect to core motor control (overall and by the type of sport).

### Correlations

Tables 3 and 4 present the Pearson correlation coefficients and associated p-values between the characteristics of core strength, core endurance and core NMC, with the four characteristics of athletic performance (overall and by the type of sport).

Overall for all sports, moderate, statistically significant correlations between IBE (strength) and the T-test, between LF strength) and the MBCT, and between AF (strength) and the T-test, VJ and MBCT were observed (Table 3). A very strong statistically significant correlation was found between LF (strength) and VJ. Because of the relatively large sample size overall, even some of the weak correlations in the range 0.27–0.39 were statistically significant.

For hockey, moderate statistically significant correlations were found between IBE (strength), LF (strength), and AF (strength) and the MBCT, and between LF (strength) and AF (strength) and the T-test (Table 4).

For netball, moderate statistically significant correlations were observed between LF (strength) and the sprint, T-test, and VJ, and between AF (endurance) and the MBCT (Table 4).

For runners, moderate statistically significant correlations were found between IBE (strength) and the VJ, between LF (strength) and the sprint, T-test, VJ and MBCT, between AF (strength) and the T-test, VJ and MBCT, between LF (endurance) and the T-test, and a strong statistically significant correlation between AF (endurance) and VJ (Table 4).

For soccer, there were moderately strong statistically significant correlations between IBE (strength) and the T-test, VJ and MBCT, between LF (strength) and VJ and MBCT, between AF (strength) and the T-test, VJ and MBCT, between AF (endurance) and the VJ, and between core NMC and the sprint, T-test and VJ (Table 4). Because of the relatively large sample size overall, even some negligible correlations in the range 0.00–0.10 were statistically significant.

For tennis, moderate statistically significant correlations were found between IBE (strength) and the T-test and MBCT, between LF (strength) and the sprint and MBCT, between AF (strength) and the sprint, T-test and MBCT, between IBE (endurance) and the sprint and MBCT, between LF (endurance) and the sprint and MBCT, and between core NMC and VJ (Table 4). A strong statistically significant correlation was found between IBE (strength) and the sprint.

Table 5 presents the results of the ANCOVA values followed by the model selection. The only selected predictor of the sprint was LF (strength). Higher LF (strength) led to lower sprint times (negative association). For the T-test, the selected predictors were IBE (endurance) (positive association), IBE (strength) and AF (strength) (negative association). For VJ, the selected predictors were AF (strength) and AF (endurance) (positive association). Furthermore, significant differences between sports with regard to VJ were observed (the p-values in Table 5 refer to the differences between tennis and the other sport codes). Finally, the only selected predictor for the MBCT was AF (strength) (positive association).

## Discussion

A well-trained athlete is expected to have general skills such as speed, agility and explosive power, in addition to sport-specific attributes. The current study’s findings were similar to the results of Sharrock et al.,^[5]^ who reported a weak relationship between core stability (as measured by the double leg lowering test) and athletic performance. Our findings are also in agreement with Nesser et al.,^[22]^ who reported a moderate correlation between core stability and sport-specific assessments.

We found that hockey players demonstrated the highest IBE, LF and AF characteristics of core strength, and tennis players the lowest. This could be attributed to the fact that the body position of a hockey player is always flexed at the lumbar spine, with combined rotational movements that require good core strength during various hitting and pushing techniques.^[23]^ Zingaro^[24]^ proposed that the core and upper extremity muscles are responsible for 54% of force production when delivering in a tennis serve. Moreover, it has been found that the speed of shoulder movement when serving can be up to 76 kilometres an hour, which could imply that most of a serve’s explosive power in tennis players originates from the shoulder and not the core. Despite inconsistent findings, researchers are of the opinion that different sporting codes require different functions of core strength.

Runners had the highest IBE, LF and AF characteristics of core endurance, and netball players the lowest. Tong et al.^[25]^ noted that core muscle fatigue may limit running endurance. Clark et al.^[26]^ reported that improved core endurance reduced overall running times in high school cross-country runners. We concur with previous literature^[25,26]^ that runners require core endurance for improved athletic performance, as a positive correlation was found between characteristics for core endurance and the sprint and T-test. Optimal performance in netball depends on the interaction between several fundamental factors relating to the balance, agility and explosive power of players.^[27]^ Hence, muscle endurance is not the most relevant component in training interventions for netball players. In addition, netball players depend more on the eccentric strength of the quadriceps when cutting and landing, rather than the core musculature,^[28]^ which could explain why the netball players in this study showed the lowest mean values for core endurance. This is supported by the fact that in this study netball players had lower core NMC (grade 1) than tennis players (grade 3) and runners (grade 4). Similar to our findings, Venter et al.^[28]^ reported that netball players rely more on lower extremity strength for cutting and landing movements than the core musculature.

Diverse views on the significance of core stability in sporting performance still exist. No studies reviewed for this research used our battery of tests to assess the different components of core stability (strength, endurance and neuromuscular control), as well as its relationship to different sports (hockey, netball, runners, soccer and tennis). Therefore, no meaningful comparisons with other studies reported in the literature could be made in this regard.

Potential limitations of this study are that only five sporting codes were examined. These sporting codes’ specific techniques and skills might not be representative of all sports. Only female athletes of the UFS participated, while male athletes may yield different results from female athletes. Athletes were assessed during different times in the conditioning season, and differences in conditioning training programmes might have influenced the results. The lack of gold standard tests to assess the strength, endurance and neuromuscular components of core stability could also be a limitation. Sport-specific assessments should be considered to assess core stability. The athletic performance tests did not account for the specific demands of the different sporting codes. Core stability and athletic performance are complex concepts, with multiple factors playing a role in both. Future research on core stability and athletic performance, with specific attention to the demands of different sporting codes, would benefit both the sport and rehabilitation sectors.

## Conclusion

This study found correlations between core stability and athletic performance, even though many correlations were only weak or moderately weak. Different sporting codes seem to require different components of core stability. When these sporting codes are considered separately, there were moderately strong correlations between core stability and its sub-groups and athletic performance tests.

Therefore, core stability can be considered an important modality when trying to improve athletic performance, but should not be the primary focus of exercise training programmes. The findings of this study can equip athletes, coaches, conditioning staff and rehabilitation specialists to better design exercise training programmes by implementing sport-specific modalities into programmes that duplicate the demands of the respective sporting codes.

## Figures and Tables

**Table 1:** Descriptive statistics for participants’ core strength and core endurance: overall and by type of sport

	All sports (n=83)	Hockey (n=24)	Netball (n=16)	Runner (n=15)	Soccer (n=17)	Tennis (n=11)
**IBE (strength) (N)**						
Mean	1002	1056	1037	1024	1017	783
Minimum	687	750	687	757	748	745
Maximum	1517	1422	1517	1303	1517	540
**IBE (endurance) (s)**						
Mean	149.7	143.9	97.8	198.8	140.2	185.9
Minimum	50.0	50.0	55.1	84.0	72.0	135.0
Maximum	149.7	143.9	97.8	198.8	140.2	185.9
**LF (strength) (N)**						
Mean	799	858	799	773	824	667
Minimum	565	586	639	565	625	625
Maximum	1082	1082	1018	984	1018	701
**LF (endurance) (s)**						
Mean	67.6	71.2	45.0	91.0	62.8	68.4
Minimum	13.0	19.0	13.0	37.5	17.3	45.9
Maximum	181.0	145.0	181.0	141.0	91.0	140.0
**AF (strength) (N)**						
Mean	897	958	906	859	941	736
Minimum	646	646	684	687	695	690
Maximum	1300	1274	1300	1188	1214	769
**AF (endurance) (s)**						
Mean	149.2	158.0	101.3	203.7	135.2	146.8
Minimum	40.0	50.0	40.0	83.0	71.0	71.0
Maximum	376.0	354.0	271.0	376.0	225.0	195.0

IBE, isometric back extension; LF, lateral flexion; AF, abdominal flexion; N, Newton; s, seconds.

**Table 2:** Descriptive statistics for core motor/neuromuscular control (NMC) grading: overall and by type of sport

Team	Statistic	NMC grading					Total

		1	2	3	4	5	
**All sports**	Frequency	13	17	31	21	1	83
	Percent	16	21	37	25	1	
**Hockey**	Frequency	3	3	10	8	0	24
	Percent	13	13	42	33	0	
**Netball**	Frequency	9	5	1	1	0	16
	Percent	56	31	6	6.3	0	
**Runner**	Frequency	0	2	6	6	1	15
	Percent	0	13	40	40	7	
**Soccer**	Frequency	1	6	7	3	0	17
	Percent	6	35	41	18	0	
**Tennis**	Frequency	0	1	7	3	0	11
	Percent	0	9	64	27	0	

**Table 3:** Pearson correlation between core strength, core endurance and core motor control tests with athletic performance tests: all sports (n=83)

Characteristic of core stability	Statistic	Athletic performance tests			

		Sprint	T-test	VJ	MBCT
**IBE (strength)**	Correlation	−0.13	**−0.44**	0.38	0.36
	p-value	0.26	0.00*	0.00*	0.00*
	95% CI	−0.33 to 0.09	−0.60 to −0.25	0.17 to 0.55	0.15 to 0.53

**IBE (endurance)**	Correlation	−0.16	0.10	−0.14	−0.09
	p-value	0.16	0.37	0.21	0.42
	95% CI	−0.36 to 0.06	−0.12 to 0.31	−0.34 to 0.08	−0.30 to 0.13

**LF (strength)**	Correlation	−0.10	−0.39	**0.90**	**0.51**
	p-value	0.35	0.00*	0.00*	0.00*
	95% CI	−0.31 to 0.12	−0.55 to −0.18	0.19 to 0.56	0.33 to 0.65

**LF (endurance)**	Correlation	−0.28	−0.06	0.05	0.09
	p-value	0.01*	0.59	0.65	0.43
	95% CI	−0.47 to −0.07	−0.27 to 0.16	−0.17 to 0.26	−0.13 to 0.30

**AF (strength)**	Correlation	−0.05	**−0.44**	**0.44**	**0.48**
	p-value	0.62	0.00*	0.00*	0.00*
	95% CI	−0.27 to 0.16	−0.60 to −0.25	0.24 to 0.60	0.30 to 0.63

**AF (endurance)**	Correlation	−0.35	−0.18	0.34	0.27
	p-value	0.00*	0.10	0.00*	0.01*
	95% CI	−0.52 to −0.14	−0.38 to 0.04	0.13 to 0.51	0.06 to 0.46

**NMC**	Correlation	−0.32	−0.12	−0.05	−0.05
	p-value	0.00*	0.27	0.68	0.65
	95% CI	−0.50 to −0.11	−0.33 to 0.10	−0.26 to 0.17	−0.26 to 0.17

* Indicates statistically significant (p<0.05).

Bold values indicates moderate, moderately strong and strong correlations. VJ, vertical jump; MBCT, medicine ball chest throw; IBE, isometric back extension; LF, lateral flexion; AF, abdominal flexion; NMC, neuromuscular control.

**Table 4:** Pearson correlation between core strength, core endurance and core motor/neuromuscular control tests with athletic performance tests

Dependent variable	Statistic	Sprint	T-test	VJ	MBCT
**HOCKEY (n=24)**					
**IBE (strength)**	Correlation	0.05	−0.38	0.33	**0.54**
	p-value	0.83	0.07	0.12	0.01*
	95% CI	−0.36 to 0.44	−0.68 to 0.03	−0.09 to 0.64	0.16 to 0.77
**IBE (endurance)**	Correlation	−0.26	−0.19	0.15	0.05
	p-value	0.22	0.37	0.49	0.83
	95% CI	−0.60 to 0.16	−0.55 to 0.23	−0.27 to 0.52	−0.36 to 0.44
**LF (strength)**	Correlation	−0.12	**−0.50**	0.37	**0.61**
	p-value	0.58	0.01*	0.07	0.01*
	95% CI	−0.50 to 0.30	−0.74 to −0.10	−0.04 to 0.67	0.26 to 0.81
**LF (endurance)**	Correlation	−0.29	−0.34	−0.00	−0.00
	p-value	0.17	0.10	0.99	0.99
	95% CI	−0.62 to 0.13	−0.65 to 0.08	−0.41 to 0.40	−0.41 to 0.40
**AF (strength)**	Correlation	0.12	**−0.41**	0.31	**0.61**
	p-value	0.55	0.05*	0.14	0.00*
	95% CI	−0.29 to 0.50	−0.69 to 0.01	−0.12 to 0.63	0.26 to 0.81
**AF (endurance)**	Correlation	−0.25	−0.21	0.15	0.18
	p-value	0.24	0.32	0.48	0.40
	95% CI	−0.59 to 0.18	−0.56 to 0.21	−0.27 to 0.52	−0.25 to 0.54
**NMC**	Correlation	−0.24	−0.11	0.04	−0.14
	p-value	0.27	0.61	0.85	0.50
	95% CI	−0.58 to 0.19	−0.49 to 0.31	−0.37 to 0.44	−0.52 to 0.28

**NETBALL (n=16)**					
**IBE (strength)**	Correlation	0.16	−0.13	−0.48	−0.42
	p-value	0.55	0.64	0.06	0.10
	95% CI	−0.37 to 0.60	−0.58 to 0.40	−0.78 to 0.04	−0.75 to 0.11
**IBE (endurance)**	Correlation	0.30	0.17	−0.18	−0.01
	p-value	0.26	0.53	0.52	0.97
	95% CI	−0.24 to 0.69	−0.36 to 0.61	−0.61 to 0.36	−0.50 to 0.49
**LF (strength)**	Correlation	**0.62**	**0.56**	**−0.51**	−0.30
	p-value	0.01*	0.02*	0.05*	0.26
	95% CI	0.16 to 0.85	0.07 to 0.82	−0.79 to 0.00	−0.69 to 0.24
**LF (endurance)**	Correlation	−0.11	−0.11	−0.08	−0.06
	p-value	0.68	0.68	0.76	0.81
	95% CI	−0.57 to 0.41	−0.57 to 0.41	−0.55 to 0.43	−0.54 to 0.45
**AF (strength)**	Correlation	0.27	0.24	−0.06	−0.09
	p-value	0.32	0.37	0.82	0.75
	95% CI	−0.27 to 0.67	−0.30 to 0.65	−0.54 to 0.45	−0.56 to 0.43
**AF (endurance)**	Correlation	−0.20	−0.38	0.19	**0.44**
	p-value	0.45	0.15	0.48	0.09
	95% CI	−0.63 to 0.33	−0.73 to 0.16	−0.34 to 0.62	−0.09 to 0.76
**NMC**	Correlation	−0.06	−0.04	−0.25	−0.30
	p-value	0.81	0.88	0.36	0.25
	95% CI	−0.54 to 0.45	−0.52 to 0.47	−0.66 to 0.29	−0.69 to 0.24

**RUNNING (n=15)**					
**IBE (strength)**	Correlation	−0.36	−0.33	**0.40**	0.13
	p-value	0.19	0.23	0.14	0.64
	95% CI	−0.73 to 0.20	−0.71 to 0.23	−0.15 to 0.75	−0.41 to 0.60
**IBE (endurance)**	Correlation	0.33	0.15	−0.23	−0.31
	p-value	0.23	0.60	0.42	0.25
	95% CI	−0.23 to 0.72	−0.40 to 0.61	−0.66 to 0.33	−0.71 to 0.24
**LF (strength)**	Correlation	**−0.41**	**−0.44**	**0.56**	**0.63**
	p-value	0.12	0.10	0.03*	0.01*
	95% CI	−0.76 to 0.14	−0.77 to 0.11	0.04 to 0.83	0.15 to 0.86
**LF (endurance)**	Correlation	0.21	**0.49**	0.11	0.00
	p-value	0.46	0.07	0.07	0.99
	95% CI	−0.35 to 0.65	−0.05 to 0.79	−0.43 to 0.59	−0.51 to 0.51
**AF (strength)**	Correlation	−0.37	**−0.55**	**0.61**	**0.52**
	p-value	0.17	0.03*	0.02*	0.05*
	95% CI	−0.74 to 0.19	−0.82 to −0.04	0.12 to 0.85	−0.01 to 0.81
**AF (endurance)**	Correlation	−0.03	−0.02	**0.71**	0.34
	p-value	0.27	0.95	0.00*	0.22
	95% CI	−0.70 to 0.26	−0.52 to 0.50	0.29 to 0.89	−0.22 to 0.72
**NMC**	Correlation	0.37	−0.14	−0.27	−0.04
	p-value	0.17	0.63	0.33	0.88
	95% CI	−0.18 to 0.74	−0.60 to 0.41	−0.68 to 0.29	−0.54 to 0.48

**SOCCER (n=17)**					
**IBE (strength)**	Correlation	−0.30	**−0.52**	**0.63**	**0.55**
	p-value	0.25	0.03*	0.01*	0.02*
	95% CI	−0.68 to 0.22	−0.79 to −0.03	0.20 to 0.85	0.07 to 0.81
**IBE (endurance)**	Correlation	0.12	0.04	−0.15	0.16
	p-value	0.65	0.89	0.57	0.53
	95% CI	−0.39 to 0.56	−0.45 to 0.51	−0.58 to 0.36	−0.35 to 0.59
**LF (strength)**	Correlation	−0.30	−0.05	**0.53**	**0.63**
	p-value	0.24	0.04*	0.03*	0.00*
	95% CI	−0.68 to 0.22	−0.78 to −0.01	0.04 to 0.80	0.19 to 0.85
**LF (endurance)**	Correlation	−0.07	−0.06	−0.08	0.27
	p-value	0.78	0.83	0.75	0.29
	95% CI	−0.53 to 0.42	−0.52 to 0.44	−0.54 to 0.42	−0.25 to 0.66
**AF (strength)**	Correlation	−0.34	**−0.56**	**0.58**	**0.46**
	p-value	0.17	0.02*	0.01*	0.06
	95% CI	−0.70 to 0.17	−0.81 to −0.09	0.12 to 0.83	−0.04 to 0.76
**AF (endurance)**	Correlation	−0.09	−0.26	**0.41**	0.15
	p-value	0.74	0.31	0.10	0.56
	95% CI	−0.54 to 0.41	−0.66 to 0.26	−0.10 to 0.74	−0.36 to 0.58
**NMC**	Correlation	−0.55	−0.56	**0.44**	0.36
	p-value	0.02*	0.02*	0.08	0.15
	95% CI	−0.81 to −0.08	−0.81 to −0.09	−0.07 to 0.75	−0.16 to 0.71

**TENNIS (n=11)**					
**IBE (strength)**	Correlation	**−0.74**	**−0.59**	0.25	**0.58**
	p-value	0.01*	0.05	0.45	0.06
	95% CI	−0.92 to −0.21	−0.87 to 0.04	−0.42 to 0.73	−0.05 to 0.87
**IBE (endurance)**	Correlation	**−0.67**	−0.31	0.28	**0.48**
	p-value	0.03*	0.34	0.40	0.13
	95% CI	−0.90 to −0.08	−0.76 to 0.37	−0.40 to 0.75	−0.19 to 0.83
**LF (strength)**	Correlation	**−0.56**	−0.15	0.20	**0.52**
	p-value	0.07	0.66	0.55	0.10
	95% CI	−0.86 to 0.08	−0.78 to 0.50	−0.46 to 0.71	−0.14 to 0.85
**LF (endurance)**	Correlation	**−0.43**	−0.35	0.22	0.58
	p-value	0.19	0.30	0.52	0.06
	95% CI	−0.81 to 0.25	−0.78 to 0.33	−0.45 to 0.72	−0.05 to 0.87
**AF (strength)**	Correlation	**−0.55**	**−0.43**	−0.19	**0.40**
	p-value	0.08	0.19	0.58	0.22
	95% CI	−0.86 to 0.10	−0.81 to 0.25	−0.70 to 0.47	−0.28 to 0.80
**AF (endurance)**	Correlation	−0.32	0.02	0.25	0.24
	p-value	0.34	0.95	0.46	0.48
	95% CI	−0.76 to 0.36	−0.59 to 0.61	−0.42 to 0.73	−0.43 to 0.73
**NMC**	Correlation	−0.35	−0.09	**0.49**	0.23
	p-value	0.29	0.80	0.13	0.49
	95% CI	−0.78 to 0.33	−0.65 to 0.54	−0.18 to 0.83	−0.44 to 0.73

* Indicates statistically significant (p<0.05).

Bold values indicates moderate, moderately strong and strong correlations. VJ, vertical jump; MBCT, medicine ball chest throw; IBE, isometric back extension; LF, lateral flexion; AF, abdominal flexion; NMC, neuromuscular control; CI, confidence interval

**Table 5:** ANCOVA with model selection: relationship between athletic performance and core endurance, core strength and core neuromuscular control

Dependent variable	Independent variable	Estimate	Standard error	p-value
**Sprint**	Intercept	4.439	0.282	
	LF (strength)	−0.0009	0.0002	0.00*

**T-test**	Intercept	4.740	2.432	
	IBE (strength)	−0.0008	0.0003	0.03*
	AF (strength)	−0.003	0.0005	0.00*
	IBE (endurance)	0.003	0.001	0.02*

**VJ**	Intercept	39.135	3.900	
	Hockey	−3.395	1.490	0.03*
	Netball	1.7548	1.427	0.22
	Runner	4.164	1.267	0.00*
	Soccer	3.217	1.128	0.01*
	AF (strength)	0.009	0.003	0.00*
	AF (endurance)	0.017	0.007	0.01*

**MBCT**	Intercept	−1.266	1.286	
	AF (strength)	0.001	0.0003	0.00*

* Indicates statistically significant (p<0.05).

ANCOVA, analysis of covariance; VJ, vertical jump; MBCT, medicine ball chest throw; LF, lateral flexion; IBE, isometric back extension; AF, abdominal flexion.
